# KBase: The United States Department of Energy Systems Biology Knowledgebase

**DOI:** 10.1038/nbt.4163

**Published:** 2018-07-06

**Authors:** Adam P Arkin, Robert W Cottingham, Christopher S Henry, Nomi L Harris, Rick L Stevens, Sergei Maslov, Paramvir Dehal, Doreen Ware, Fernando Perez, Shane Canon, Michael W Sneddon, Matthew L Henderson, William J Riehl, Dan Murphy-Olson, Stephen Y Chan, Roy T Kamimura, Sunita Kumari, Meghan M Drake, Thomas S Brettin, Elizabeth M Glass, Dylan Chivian, Dan Gunter, David J Weston, Benjamin H Allen, Jason Baumohl, Aaron A Best, Ben Bowen, Steven E Brenner, Christopher C Bun, John-Marc Chandonia, Jer-Ming Chia, Ric Colasanti, Neal Conrad, James J Davis, Brian H Davison, Matthew DeJongh, Scott Devoid, Emily Dietrich, Inna Dubchak, Janaka N Edirisinghe, Gang Fang, José P Faria, Paul M Frybarger, Wolfgang Gerlach, Mark Gerstein, Annette Greiner, James Gurtowski, Holly L Haun, Fei He, Rashmi Jain, Marcin P Joachimiak, Kevin P Keegan, Shinnosuke Kondo, Vivek Kumar, Miriam L Land, Folker Meyer, Marissa Mills, Pavel S Novichkov, Taeyun Oh, Gary J Olsen, Robert Olson, Bruce Parrello, Shiran Pasternak, Erik Pearson, Sarah S Poon, Gavin A Price, Srividya Ramakrishnan, Priya Ranjan, Pamela C Ronald, Michael C Schatz, Samuel M D Seaver, Maulik Shukla, Roman A Sutormin, Mustafa H Syed, James Thomason, Nathan L Tintle, Daifeng Wang, Fangfang Xia, Hyunseung Yoo, Shinjae Yoo, Dantong Yu

**Affiliations:** 1 Department of Bioengineering, University of California, Berkeley, California USA; 2 Environmental Genomics and Systems Biology Division, E.O. Lawrence Berkeley National Laboratory, Berkeley, California USA; 3 Biosciences Division, Oak Ridge National Laboratory, Oak Ridge, Tennessee USA; 4 Mathematics and Computer Science Division, Argonne National Laboratory, Argonne, Illinois USA; 5 Computer Science Department and Computation Institute, University of Chicago, Chicago, Illinois USA; 6 Computing, Environment, and Life Sciences Directorate, Argonne National Laboratory, Argonne, Illinois USA; 7 Biology Department, Brookhaven National Laboratory, Upton, New York USA; 8 Cold Spring Harbor Laboratory, Cold Spring Harbor, New York USA; 9 Computational Research Division, E.O. Lawrence Berkeley National Laboratory, Berkeley, California USA; 10 Berkeley Institute for Data Science, University of California, Berkeley, California USA; 11 Department of Statistics, University of California, Berkeley, California USA; 12 National Energy Research Scientific Computing Center, E.O. Lawrence Berkeley National Laboratory, Berkeley, California USA; 13 Department of Biology, Hope College, Holland, Michigan USA; 14 Department of Plant and Microbial Biology, University of California, Berkeley, California USA; 15 Department of Computer Science, Hope College, Holland, Michigan USA; 16 Computation Institute, University of Chicago, Chicago, Illinois USA; 17 Program in Computational Biology and Bioinformatics, Yale University, New Haven, Connecticut USA; 18 Department of Plant Pathology and Genome Center, University of California, Davis, Davis, California USA; 19 Joint BioEnergy Institute, Lawrence Berkeley National Laboratory, Berkeley, California USA; 20 Department of Microbiology, University of Illinois at Urbana-Champaign, Urbana, Illinois USA; 21 Department of Plant Sciences, University of Tennessee, Knoxville, Tennessee USA; 22 Department of Mathematics, Hope College, Holland, Michigan USA; 23 Computer Science and Math, Computer Science Initiative, Brookhaven National Laboratory, Upton, New York USA; 24 Present Address: Present addresses: Department of Bioengineering and Carl R. Woese Institute for Genomic Biology, University of Illinois at Urbana-Champaign, Urbana, Illinois, USA (S.M.); Department of Statistics, University of California, Berkeley, California, USA (F.P.); New York University Shanghai Campus, Pudong, Shanghai, China (G.F.); Department of Plant Pathology, Kansas State University, Manhattan, Kansas, USA (F.H.); Insilicogen. Inc., Giheung-gu, Yongin-si, Gyeonggi-do, Korea (T.O.); Department of Computer Science, Johns Hopkins University, Baltimore, Maryland, USA (S.R., M.C.S.); Memorial Sloan Kettering Cancer Center, New York, New York, USA (M.H.S.); Dordt College, Sioux Center, Iowa, USA (N.L.T.); Department of Biomedical Informatics, Stony Brook University, Stony Brook, New York, USA (D.W.); Martin Tuchman School of Management, New Jersey Institute of Technology, Newark, New Jersey, USA (D.Y.)., ,

**Keywords:** Software, Data integration, Computational platforms and environments, Databases

To the Editor:

Over the past two decades, the scale and complexity of genomics technologies and data have advanced from sequencing genomes of a few organisms to generating metagenomes, genome variation, gene expression, metabolites, and phenotype data for thousands of organisms and their communities. A major challenge in this data-rich age of biology is integrating heterogeneous and distributed data into predictive models of biological function, ranging from a single gene to entire organisms and their ecologies. The US Department of Energy (DOE) has invested substantially in efforts to understand the complex interplay between biological and abiotic processes that influence soil, water, and environmental dynamics of our biosphere. The community that has grown around these efforts recognizes the need for scientists of diverse backgrounds to have access to sophisticated computational tools that enable them to analyze complex and heterogeneous data sets and integrate their data and results effectively with the work of others. In this way, new data and conclusions can be rapidly propagated across existing, related analyses and easily discovered by the community for evaluation and comparison with previous results^1,2,3^.

Here we present the DOE Systems Biology Knowledgebase (KBase, http://kbase.us), an open-source software and data platform that enables data sharing, integration, and analysis of microbes, plants, and their communities. KBase maintains an internal reference database that consolidates information from widely used external data repositories. This includes over 90,000 microbial genomes from RefSeq^4^, over 50 plant genomes from Phytozome^5^, over 300 Biolog media formulations^6^, and >30,000 reactions and compounds from KEGG^7^, BIGG^8^, and MetaCyc^9^. These public data are available for integration with user data where appropriate (e.g., genome comparison or building species trees). KBase links these diverse data types with a range of analytical functions within a web-based user interface. This extensive community resource facilitates large-scale analyses on scalable computing infrastructure and has the potential to accelerate scientific discovery, improve reproducibility, and foster open collaboration (Supplementary Note 1).

Although similar integrative tools exist (Supplementary Note 2), no other open platform shares all KBase's features, which include the following: (i) comprehensive support for data provenance and analysis reproducibility; (ii) a flexible system for sharing data and workflows; (iii) an integrated database of genomes and biochemistry; (iv) a point-and-click interface that enables users to build, store, run, and share complex scientific analyses of fully integrated data; (v) built-in support for the use of custom code interleaved with point-and-click apps; and (vi) a software development kit that enables external developers to add applications to KBase (Supplementary Table 1). KBase has a suite of scientific applications that enables users to build and share sophisticated workflows. For example, a user can predict species interactions from metagenomic data by assembling raw reads, binning assembled contigs by species, annotating genomes, aligning RNA-seq reads, and reconstructing and analyzing individual and community metabolic models. KBase supports numerous branch points, alternative pipelines, alternative entry points, and internal curation loops that facilitate a wide range of scientific analyses, some of which are not available elsewhere (e.g., merging individual metabolic models into community models and using these to predict interspecies interactions). Although KBase was developed to support analysis of microbes, plants, and their communities, it is potentially applicable to any area of science. There is, however, a policy on use restriction for projects that require HIPAA compliance.

KBase's primary user interface, the Narrative Interface, provides a user experience distinct from other analysis platforms available today, although it shares some common features with a few other systems (Supplementary Note 2). From this interface, which is built on the Jupyter^10,11^ platform, users can upload their private data, search and retrieve extensive public reference data, access data shared by others, share their data with others, select and run applications on their data, view and analyze the results from those applications, and record their thoughts and interpretations along with the analysis steps. These activities take place within a point-and-click 'notebook' environment (Fig. 1). When a user begins a new computational experiment in KBase, they create a new 'notebook' (referred to as a Narrative in KBase) to hold this experiment. Every action performed by a user appears as a 'cell' in the Narrative. App cells show the chosen input parameters for the application and the results of the analysis. Markdown cells allow users to add formatted text and figures to a Narrative to describe the thought process behind the scientific workflow being crafted.

**Figure 1:**
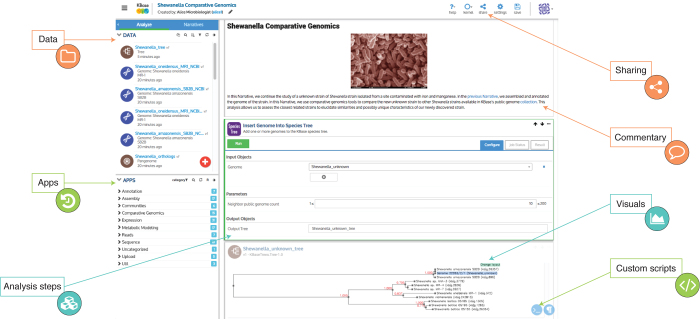
KBase Narratives. A Narrative is an interactive, dynamic, and persistent document created by users that promotes open, reproducible, and collaborative science.

A finished Narrative is a precise record of everything the authors did to complete their analysis. Although Narratives are private by default, users may choose to make their Narratives public, or share them with other individual users. This recording of a user's KBase activities within a sharable Narrative is a central pillar of KBase's support for reproducible, transparent research (Supplementary Note 1). Once a Narrative has been shared or made public, other users can copy the Narrative and rerun it on their own data, or modify it to suit their scientific needs. Thus, public Narratives serve as resources for the user community by capturing valuable data sets, associated computational analyses, and scientific context describing the rationale behind a scientific study in a form that is immediately reproducible and reusable. A growing number of public Narratives are available in KBase, some of which are showcased in the Narrative Library (https://kbase.us/narrative-library/).

The data model in KBase is fundamental to supporting reproducibility and collaboration. KBase is built upon an object-oriented data model where each object instance is automatically versioned and linked to provenance information describing how it was generated. Each data object is also associated with the specific Narrative in which it was uploaded or generated. When a Narrative is shared or copied, all its input and output data are shared or copied with it. Currently supported data types include reads, contigs, genomes, metabolic models, growth media, RNA-seq, expression, growth phenotype data, and flux balance analysis solutions. This set of types can be extended to support new apps and functionality.

Many existing systems (Supplementary Note 2) provide similar support for object-level sharing and provenance, but these systems operate on raw files only, without integration into a common data model. In KBase, objects are not simple files—they are explicitly defined and validated data structures, within which associated objects are linked to one another. For example, a metabolic model IS linked to its associated genome, which is linked to its associated taxonomy. This data model enhances interoperability by requiring apps to operate on a common data representation. Furthermore, it enhances awareness of interdependence so users can be notified when an object on which an analysis is based has been updated, and it will ultimately enable data discovery and meta-analysis across the KBase platform.

Presently, KBase has over 160 apps (https://narrative.kbase.us/#appcatalog) offering diverse scientific functionality for (meta)genome assembly, contig binning, genome annotation, sequence homology analysis, tree building, comparative genomics, metabolic modeling, community modeling, gap-filling, RNA-seq processing, and expression analysis (see Supplementary Note 2 for references). Apps interoperate seamlessly to enable a range of scientific workflows (Fig. 2). For reproducibility, all apps in KBase are containerized in versioned Docker modules, enabling a user to run any version at any time.

**Figure 2:**
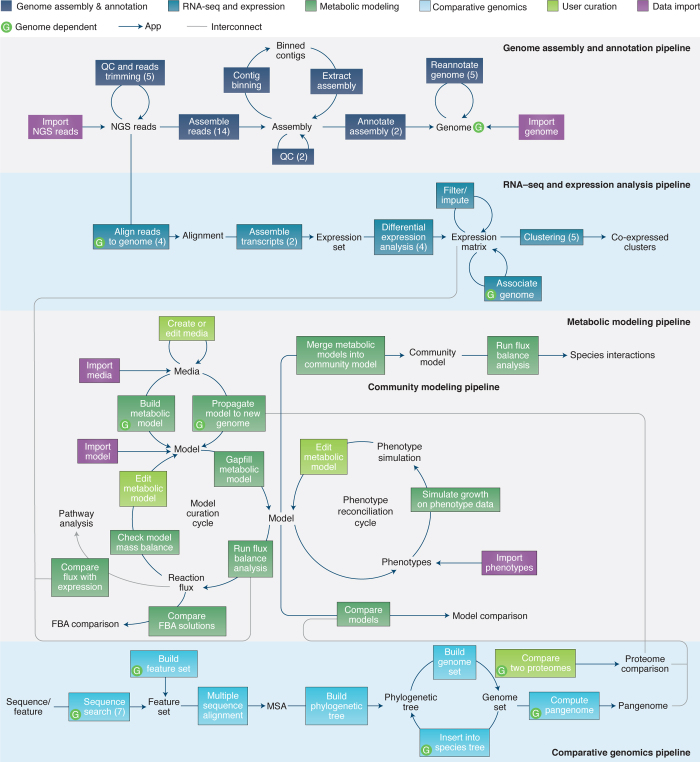
Major workflows and data types in KBase. The unboxed labels represent data types, while each colored box represents a single app. The box colors signify the category of functionality, and the numbers in parentheses indicate the number of alternative apps that implement each function. Apps that require a genome data type as input are marked with a green 'G' icon. For more information see http://kbase.us/apps/.

In addition to running apps, users can create and run blocks of code within a Narrative using “code cells.” KBase has an application-programming interface (API) that allows users to call any KBase app programmatically from within these code cells. This enables users to, for example, run large-scale studies in KBase (e.g., building thousands of models at once) by using loops within a code cell (Supplementary Note 1; Fig. 1). Users can also leverage the flexibility of code cells to add custom analysis steps that are not yet available as KBase apps.

Although there are other systems that allow users to create workflows consisting of a series of analysis tool runs and code blocks, the app functionality in KBase differs from these systems in several ways (Supplementary Note 2). Currently, KBase's capabilities for community model reconstruction, plant model reconstruction, community model gap-filling, and expression data model integration are unique to the KBase platform (Supplementary Note 1; Fig. 2).

KBase was designed to be an extensible community resource. This extensibility is supported by the KBase Software Development Kit (SDK), which is a set of command-line tools and a web interface that enable any developer to build, test, register, and deploy new or existing software as KBase apps, thereby extending the platform's scientific capabilities. All software contributed to the central KBase software repository must adhere to a standard open-source license (https://opensource.org/licenses). Information about the app developer is maintained in the documentation for that app so credit can be given to the contributor. Data provenance, job management, usage logging, and app versioning are handled automatically by the platform, allowing developers with minimal training to package scientific tools in a form that makes them accessible to users within KBase. Other existing platforms offer similar support for third-party development (Supplementary Note 2), but KBase's data model provides the additional benefit of improving interoperability of third-party applications by imposing a single data format and specification on all data types consumed or produced by each app. More information about the KBase SDK is available at https://github.com/kbase/kb_sdk/blob/master/README.md.

Many users have already discovered and applied KBase to meet their scientific needs. As of September 2017, over 3,000 users have KBase accounts, and users have created over 5,000 Narratives. These Narratives contain a total of over 250,000 data objects, or an average of 96 data objects and five apps per Narrative. Science done within Kbase, which has been published in over 30 peer-reviewed publications (Supplementary Note 1; http://kbase.us/publications), includes reconstruction of >8,000 models of core metabolism across the microbial tree of life^12^, reconstruction of semi-curated metabolic models for 773 gut microbes^13^, prediction of trophic interactions within a microbial community^14^, and reconstruction of regulons from expression data^15^.

Much of the research performed within KBase has been publicly shared as Narratives that any user can view, copy, and rerun. Through these public Narratives, scientists can rapidly follow the examples set by their peers to apply similar approaches to new data and scenarios. Thus KBase goes beyond supporting reproducible science to enabling rapid repurposing, reapplication, and extension of scientific techniques. As more users apply the system to address their scientific questions, and share their resulting Narratives, KBase will have a continually growing body of experiments, results, and scientific use cases that can be adapted and extended by other researchers.

KBase's integration of data and tools and the ease of creating and running large-scale analysis workflows have the potential to empower scientists in a broad range of application areas for systems biology, including environmental analysis, biosystems design, and human health. KBase's sharing capabilities amplify this potential by enabling scientists with differing expertise to easily work together and leverage each other's work (Supplementary Note 1; Supplementary Fig. 3).

Future development of KBase will build upon the concept of KBase as a knowledgebase. The social aspects of the platform will be enhanced, enabling scientists to discover colleagues with complementary talents. New data-discovery features will allow the platform to suggest data sets and Narratives that may be of interest to a particular user, based on interconnections found in the data in KBase. These features will ultimately evolve into knowledge-discovery features, enabling KBase to propose new hypotheses by making connections across the system. Information on data and code availability can be found in Supplementary Note 3.

*Editor's note: This article has been peer-reviewed.*

**Author contributions**

A.P.A., R.W.C., C.S.H., R.L.S., S.M., P.D., D.W., and F.P. developed the concept and vision. A.P.A., C.S.H., R.L.S., S.C., M.W.S., M.L.H., W.J.R., D.M.O., S.Y.C., T.S.B., D.C., D.G., J.B., A.A.B., B.P.B., S.E.B., C.C.B., J.M.C., J.C., R.C., N.C., J.J.D., M.D.J., S.D., A.G., F.H., M.P.J., K.P.K., F.M., P.S.N., R.O., E.P., S.P., G.A.P., S.R., P.R., S.M.D.S., M.S., R.A.S., M.H.S., J.T., F.X., H.Y., S.J.Y., and D.Y. designed and developed the system. R.W.C., N.L.H., R.T.K., S.K., M.M.D., E.M.G., D.C., D.J.W., B.H.A., B.H.D., E.D., I.D., J.N.E., G.F., J.P.F., P.M.F., W.G., M.G., J.G., R.J., S.N.K., V.K., M.L.L., M.M., T.Y.O., G.J.O., B.P., S.S.P., P.C.R., M.C.S., N.L.T., and D.F.W. developed, documented and conducted testing and validation. A.P.A., C.S.H., and N.L.H. drafted the manuscript. N.L.H., H.L.H., B.H.A., M.M.D., M.P.J., A.A.B., J.M.C., D.C., R.O., B.H.D., N.L.T., S.M., P.C.R., M.D.J., and V.K. revised the manuscript and provided important intellectual content. J.B., M.P.J., J.M.C., V.K., J.N.E., J.P.F., S.M.D.S. provided content for the supplementary information. AP.A., R.W.C., C.S.H., and N.L.H. reviewed and approved the final version to be published.

## Supplementary information

Supplementary Text and Figures Supplementary Figures 1–3, Supplementary Table 1, and Supplementary Notes 1–3 (PDF 566 kb)

Life Sciences Reporting Summary (PDF 98 kb)
