# Supplementary material for: KBase: The United States Department of Energy Systems Biology Knowledgebase
Source: Nat Biotechnol. 2018 Jul 6;36(7):566–9. doi: 10.1038/nbt.4163 (PMC6870991; doi:10.1038/nbt.4163)
Supplement: Supplementary Text and Figures — Supplementary Figures 1–3, Supplementary Table 1, and Supplementary Notes 1–3 (PDF 566 kb) [file 41587_2018_BFnbt4163_MOESM12_ESM.pdf]

Supplementary Notes

Supplementary Note 1. Examples of Use of KBase Infrastructure and Tools

The KBase platform has been utilized by a variety of scientific groups, with citations in over 30 peer-reviewed publications. These publications cover a range of topics and demonstrate the novel ways scientists are applying tools within KBase to their research. Several of these studies have publicly shared KBase Narratives associated with them. These serve as a mechanism to show how the KBase platform was applied to perform the described work as well as a means of releasing the data produced in the published analysis. The KBase website maintains a curated list of published Narratives at http://www.kbase.us/narrative-library/. Here we will review a selection of these research Narratives that demonstrate many (but not all) of the workflows and capabilities available in the KBase platform (see Figure 2 in the main manuscript).

1A. Reconstruction of 8000 Models of Core Metabolism Across the Microbial Tree of Life

Link: https://narrative.kbase.us/narrative/ws.20186.obj.18

In a recent publication in *BMC Genomics*[1] researchers developed a framework in KBase to reconstruct and analyze core metabolic models (CMMs). Core metabolic models representing 48 major phylogenetic microbial groups were constructed based on a core model template consisting of a highly curated set of biochemical reactions derived from a diverse set of model organisms, and about 200 unique reactions were selected from this set, comprising 12 key energy biosynthesis pathways linked to central metabolism and variations of bacterial electron transport chains. This framework was applied in KBase to build core models for over 8000 prokaryotic genomes that span the prokaryotic tree of life. The authors used CMMs to determine: (i) accurate ATP yields based on different growth/environmental conditions; (ii) ETC variations and respiration types; (iii) ability to produce fermentation products; (iv) presence and absence of classical biochemical pathways in central metabolism; and (v) ability to produce key metabolic pathway intermediates in central metabolism which are precursors of essential biomass components of the cell.

The Narrative associated with this work utilizes the core model reconstruction, gapfilling, and flux balance analysis (FBA) workflows in KBase. It also demonstrates the capacity of KBase to perform large-scale analyses. The authors used a *code cell* to programmatically run the model reconstruction, gapfilling, and FBA workflow on over 8000 genomes (Figure 1). Code cells make it possible to run any KBase App programmatically from within a Narrative. One can write a code cell in the Python programming language within a Narrative to run a single app or an entire pipeline of apps. While code cells require knowledge of Python programming to write, Narratives with useful code cells can be shared with any user, who can re-run the code cells with ease. This work also inspired the addition of a new batch app for model reconstruction called *Build*

Multiple Metabolic Models. With this new app, it is no longer necessary to use a code cell to build thousands of models. Such batch apps demonstrate a further mechanism beyond code cells through which the KBase platform can be used to conduct large studies. To download the 8000+ models from KBase in SBML form, the authors used the *Bulk Download Modeling Objects* app, which permits bulk download of any supported type modeling object.

1B. Identifying Violacein Synthesis Genes in a New Isolate Genome

Link: https://narrative.kbase.us/narrative/ws.21546.obj.1

With its many apps for microbial genome assembly and annotation, KBase is an ideal platform for the analysis of new isolate genomes. This is demonstrated in a recent *Genome Announcement* publication by Romy Chakraborty and colleagues[2]. In this study, groundwater samples from the ORNL FRC site were collected from multiple wells, where researchers identified a *Janthinobacterium* isolate that produces violacein. Violacein is a naturally-occurring bis-indole pigment with antibiotic (antibacterial, anti-fungal and anti-tumor) properties. The Chakraborty team loaded their isolate reads into KBase, performing QC and assembly. They next applied both the Prokka[3] and RAST[4] annotation tools to the assembled genome, with Prokka calling 5531 genes, while RAST called 5998. They also ran OrthoMCL[5] to compare the two alternative annotations side by side. From this analysis, the researchers determined that both RAST and Prokka properly annotated 4 out of 5 genes in the violacein biosynthesis pathway. However, these algorithms differed in the specific violacein gene they missed. RAST missed VioC, while Prokka missed VioE. By combining both sets of annotations, the researchers were able to identify and confirm the presence of all violacein genes in their isolate of interest. The Chakraborty team's analysis is captured in the linked Narrative, including their thought process at each step. This study demonstrates the value of having multiple apps for some of the key steps of the isolate analysis pipeline. During genome assembly and genome annotation, the researchers were able to apply multiple algorithms, evaluating and comparing the results, and selecting the best-performing algorithm for each step.

1C. Predicting Trophic Interactions Within a Microbial Community

Narrative 1: https://narrative.kbase.us/narrative/ws.13807.obj.1
Narrative 2: https://narrative.kbase.us/narrative/ws.13806.obj.1
Narrative 3: https://narrative.kbase.us/narrative/ws.13838.obj.1

KBase enables researchers to study interactions between multiple species in a microbial community. In three interconnected Narratives associated with a recent cover article in the *Journal of Cellular Physiology*[6], the authors applied KBase to construct new metabolic models for a photoautotroph, *Thermosynechococcus elongatus* BP1, and a heterotroph, *Meiothermus ruber* strain A. They used these models to predict trophic interactions between species, exploring a wide range of potential methods for predicting these interactions. The authors utilized metatranscriptomic data to validate predictions and identify which prediction method performed the best (see Figure 2).

These Narratives collectively demonstrate a large fraction of the capabilities presently available in KBase, including: (i) proteome comparison; (ii) building species trees; (iii) annotating a genome; (iv) building and gapfilling a modeling; (v) propagating a model; (vi) loading expression data; (v) building a community metabolic model; (vi) running flux balance analysis; (vii) comparing flux with expression data (Figure 2); and (viii) using code cells to sift data from objects in the KBase workspace. In this case, the code cell is used to print out the trophic interactions among species in a community model based on the flux profile generated by the FBA app.

1D. Modeling Metabolic Interactions Between Cyanobacteria-Sphagnum

Link: https://narrative.kbase.us/narrative/ws.9667.obj.2

KBase has tools and datasets that are useful for analyzing plant metabolism and plant/microbe interactions. In a study published in *Plant, Cell & Environment*[7], researchers explored a mutually beneficial metabolic partnership between a moss and a bacterium, using KBase data and tools to build a merged community metabolic model in which a nitrogen-fixing diazotrophic microbe (Anabena) fixes nitrogen to allow a plant (Sphagnum) to grow. Sphagnum (peat moss), a genus of plants (Bryophyta) that associate with nitrogen-fixing diazotrophs is a quintessential ecosystem engineer. All the analysis steps were performed in KBase, culminating with merging the two models into a community model which exhibits nitrogen fixation and exchange, showing that the plant portion of the model consumes the nitrogen fixed by the microbial portion of the model, and predicting that the Sphagnum will grow more when utilizing nitrogen fixed by the microbe than when fixing nitrates on its own.

This Narrative demonstrates the plant reference genomes in KBase, as well as the plant model reconstruction pipeline. It also shows how models of any type (for example, microbial and plant models) can be merged together to form a community model.

1E. Sharing Workflows Easily with Collaborators and the General Public

Link 1: https://narrative.kbase.us/narrative/ws.18152.obj.1
Link 2: https://narrative.kbase.us/narrative/ws.18153.obj.1
Link 3: https://narrative.kbase.us/narrative/ws.18155.obj.1
Link 4: https://narrative.kbase.us/narrative/ws.18156.obj.1
Link 5: https://narrative.kbase.us/narrative/ws.18157.obj.1

These Narratives were specifically crafted to demonstrate how the KBase platform can facilitate interaction, data sharing, and collaboration between two scientists working on a common problem. The ability to share data, results, and workflows is extremely important and more or less a requirement in collaborative studies[8]. KBase facilitates copying and sharing of

collaborative data, workflows and related commentary. A user can share any Narrative that they own with other KBase users or make it publically available. Importantly, when a user shares a Narrative, they also are sharing all the data objects loaded, used, or generated within the Narrative, complete with versioning and provenance. Copying and sharing are performed in a controlled manner where users with read privileges for a Narrative can create their own copy, which they own and can edit. Users are able to quickly replicate and expand on any KBase Narrative shared with them. This approach facilitates reproducible interdisciplinary science by allowing researchers with different expertise to quickly and easily exchange data, results, methodologies, and workflows to address complex biological problems.

We demonstrate collaborative sharing of data, commentary (notes) and workflows in KBase using a series of example Narratives (Figure 3) featuring two hypothetical scientists: Alice, an experimental biologist with expertise in assembly, annotation, and comparative genomics, and Bob, a computational biologist with expertise in metabolic modeling.

Alice the experimentalist uploads raw reads that she has sequenced from a strain of *Shewanella* on which she wants to perform comparative genomic analysis in order to understand the similarities and unique features compared to phylogenetically closely related genomes. She uploads sequencing reads to KBase and then assembles and annotates the reads generating an annotated genome (Alice assembly and annotation Narrative: https://narrative.kbase.us/narrative/ws.18152.obj.1). In a separate Narrative (Alice Comparative Genomics Narrative: https://narrative.kbase.us/narrative/ws.18153.obj.1), Alice identifies genomes that are phylogenetically close to her *Shewanella* strain. She also finds growth phenotype data for *Shewanella oneidensis* MR-1, which is phylogenetically close to her strain. This inspires Alice to perform a growth phenotype array on her own strain. Alice then compares phenotype arrays of her own strain vs *S.oneidensis* MR-1 and notices many differences that she cannot explain.

In order to understand more about Biolog phenotype data, Alice contacts Bob the modeler who is able to analyze the metabolic differences between the two strains, which in turn helps Alice to interpret the Biolog phenotype data. Alice shares her Narratives with Bob, which allows Bob to copy her genomes into a new Narrative. In this Narrative (Bob build metabolic models Narrative: https://narrative.kbase.us/narrative/ws.18155.obj.1) Bob loads a published model of *S. oneidensis* MR-1, which he then propagates to Alice's genome, producing a high quality metabolic model using the published model as a template. Bob compares the two models, identifying some interesting metabolic differences. Then Bob creates a separate Narrative (Bob and Alice shared Narrative of Phenotype Data Analysis: https://narrative.kbase.us/narrative/ws.18156.obj.1) in which he imports Alice's Biolog data and simulates the data with his *Shewanella* models. He optimizes his models to fit the Biolog data and shares the results with Alice. Finally, the two scientists build another shared Narrative (Bob and Alice shared Narrative of Phenotype Data Reconciliation: https://narrative.kbase.us/narrative/ws.18157.obj.1) in which Alice improves the quality of Bob's models by curating the models further by replacing some of the gapfilled reactions with more

biologically meaningful selections, gaining a complete understanding of the differences between her strain and MR-1.

This example demonstrates how KBase can facilitate a collaborative study between scientists/groups with different but complementary expertise who are able to accomplish more together than they could individually. By saving their data, workflows along with commentary as Narratives, scientists who use KBase also enable other researchers to quickly reproduce their work with a minimum effort and also aid in further extending similar scientific research.

Supplementary Note 2. Comparison of KBase with Other Online Systems Biology Resources

KBase has over 160 apps offering diverse scientific functionality for (meta)genome assembly, contig binning[9], genome annotation[10], sequence homology analysis[5], tree building[11], comparative genomics, metabolic modeling[12], community modeling[13], gap-filling[14, 15], RNA-seq processing[16], and expression analysis[17]. Apps in KBase interoperate seamlessly to enable a range of scientific workflows. Numerous tools exist today that are similar to KBase in that they offer web-based access to a variety of systems biology workflows. Five of the most similar and widely used tools are Galaxy[18], CyVerse[19], Pathway Tools[20], BaseSpace (http://basespace.illumina.com) and GenePattern[21]. In order to highlight the ways in which KBase is different from each of these frameworks, we conducted a detailed comparison between KBase and these tools, focusing on the areas of functionality where KBase is most distinctive. Here we report on the results of our comparison, organizing our analysis into six fundamental areas: (a) user experience; (b) data model and provenance; (c) built in reference data; (d) sharing of data and workflows; (e) third-party development and custom code support; and (f) available scientific functionality.

2A. Overview of All Compared Platforms

We begin by providing some background on each of the resources selected for our comparison. The first framework we selected was Galaxy, which is a scientific workflow, data integration, and analysis platform that aims to make bioinformatics and computational biology workflows accessible to researchers without computer programming or systems administration experience. Like KBase, Galaxy is an open-source project. The project began in 2005, and it currently exists as a collaborative effort involving primarily Pennsylvania State University, Johns Hopkins University, and Oregon Health & Science University. Although Galaxy is available in many forms (described in detail at https://galaxyproject.org/citing-galaxy/), we focus our comparison on the version of Galaxy that most resembles KBase, which is the main public usegalaxy.org site.

The second framework we selected for comparison was CyVerse. CyVerse was launched by NSF in 2008 initially as the iPlant Collaborative, but in 2013, its scope was extended to all non-human life sciences, with a renaming to CyVerse in 2016. The primary mission of CyVerse is to transform broader science through data discovery. While CyVerse offers customizable and programmatic interfaces, its two primary interfaces are a point and click web interface called the

Discovery Environment, and a cloud-hosted VM app based interface called Atmosphere. As with Galaxy, we will focus most of our comparison on the web-based Discovery Environment. The CyVerse Discovery Environment provides functionality to manage data, add new algorithms and tools, and run analyses on appropriate computational resources. It also provides access to storage (user's own, shared, and public data).

The third framework we selected for comparison was the BaseSpace Sequence Hub. BaseSpace is a cloud-based, closed-source platform that provides tools to manage and analyze Illumina sequence data. Signing up for a free trial account allows users to store up to 1TB of sequence data for an indefinite period of time, as well as giving them a limited number of "compute credits". Many apps require "compute credits" to run, but free apps are also available. Like CyVerse and Galaxy, BaseSpace offers a web interface for storing and analyzing biological data, although this framework focuses primarily on sequencing data.

The fourth framework we selected for comparison was GenePattern. GenePattern provides hundreds of analytical tools for gene expression (RNA-seq and microarray), sequence variation, proteomic data, flow cytometry, and network analysis. These tools are all made available through the online Gene Pattern Notebook environment with no programming experience required. Like KBase, this notebook environment extends the Jupyter Notebook[22, 23] system, allowing researchers to create documents that interleave formatted text, graphics and other multimedia, executable code, and GenePattern analyses. GenePattern was developed at the Broad Institute starting in 2006, and it is primarily funded by the National Cancer Institute.

Given the extensive metabolic modeling tools available in KBase, we wanted to include a metabolic modeling framework in our comparison. For this, we chose Pathway Tools. Pathway Tools was developed as a means of curating and visualizing biochemical pathways and associated data for various organisms, dating all the way back to 1993. It is one of the longest lived software/database/web-server suites available. Pathway Tools supports the use of all known metabolism, in a wide taxonomical range, allowing users to create databases for bacteria, fungi, and plants. Pathway Tools integrates a web server, enabling laboratories to host their own instance of the database and publish their own data. Pathway Tools is somewhat different from the other frameworks selected for comparison in that most functionalities are only available offline through its installable software suite. Still, it is useful to include it in our comparison as it embodies many distinctive design patterns.

2B. Comparison of User Experience

KBase and all five of the platforms we selected for comparison offer a similar overall user experience. All platforms have a graphical user interface, which enables users to view data and run apps in point-and-click fashion. All platforms offer a centralized website from which users can access a canonical version of the platform. Galaxy, GenePattern, and Pathway Tools also offer a downloadable version, which can be installed and run on a user's own hardware. The KBase SDK also allows for this, but in a much less user-friendly and more limited manner.

KBase's Narrative Interface is distinct from any other analysis platforms available today, although it shares some common features with GenePattern[21]. KBase and GenePattern are both built on the Jupyter platform, allowing users to fashion multi-step analyses within online notebooks which they can then share. Both KBase and GenePattern extend Jupyter by offering users a point-and-click menu of apps that can be run within the Jupyter notebook. However, KBase is unique in also wrapping a data-layer around Jupyter, enabling users to browse and view data objects imported or generated by apps within the notebook. Pathway Tools, CyVerse, and BaseSpace are all app-centric interfaces with limited workflow support. Users select a single app, then select data to run the app on. Then the user views and shares the output of the analysis. Other point-and-click computational platforms do exist that enable users to dynamically construct workflows, including Taverna[24], XSEDE[25], myExperiment[26], Kepler[27], Pegasus[28], and Globus[29], but many of these platforms lack KBase's tight integration of tools and data, and none of these platforms offers the "story-telling" capacity of a Jupyter notebook-based interface.

Thus in the area of user experience, Galaxy and GenePattern are the most similar to KBase.

2C. Comparison of Data Model

Data model is one area where the platforms we selected for comparison vary the most. In our comparison, we found three distinct design patterns: (i) a file-based design (Galaxy, CyVerse, BaseSpace, GenePattern); (ii) a structured object-based design (KBase); and (iii) an entirely relational design (Pathway Tools).

Galaxy, CyVerse, GenePattern and BaseSpace all share very similar data frameworks in which all data is stored in the native file format uploaded by the user (e.g., FASTA, FASTQ, BAM). In these frameworks, the files are always augmented with associated metadata. This data model has several advantages: (i) upload and download are easy because there is no need to transform files into another form; (ii) sharing, provenance, and versioning are all easy because individual objects are self-contained and these features can operate on the level of each individual object; (iii) integration of files with tools is simple because tools typically operate on native file formats directly. However, this approach also has disadvantages: (i) files are only as consistent as their standards force them to be, and many files types (e.g., SBML, FASTA, GFF, GenBank) actually involve extensive variability in how they represent data; (ii) there are often many different file formats representing a single entity (e.g., FASTQ, FASTA, SRA for reads), meaning many file format conversion utilities are required and the user spends significant time transforming file formats; (iii) complex files (e.g., GenBank, FASTQ, SRA) are often treated as black boxes, meaning they lack introspection and the files are not indexed in detail; and (iv) the data that can be stored in a data type is limited by what is accommodated by its associated file formats (e.g., in COBRA, extensive data is stashed in the typed object used to represent a model, but when that typed object is converted to SBML, some of that data is lost due to limitations in the SBML file format).

The KBase data model is similar to the file-based data model in that it is also object-based. This means the KBase data model shares the largest benefit of the file-based system, which is that individual objects are self-contained and can be independently shared, versioned, and provenanced. However, instead of representing objects in their original file formats, KBase represents objects in a single standardized, typed, rigorously specified, versioned, and validated JSON-based format. The downside of this approach is that all uploaded and downloaded files must be converted to and from their associated KBase object type, which makes supporting upload and download a challenge for tool developers (the conversion process is transparent and thus has very limited impact on end-users). This conversion can be lossy if the input file and output data-type are not completely synchronized. This also makes tool integration more of a challenge, as the tool developers must add some additional code to handle the conversion to and from any KBase data type that their tool operates against. A developer may also need to add a new data type if the output of the tool must be persistent (and reused) and the type doesn't already exist. However, this approach eliminates nearly all the downsides of a file-based system: (i) types are totally standardized and consistent even if their associated files are not; (ii) there is only one representation for each fundamental type (e.g., “reads” vs SRA, FASTQ, FASTA); (iii) types can be summarized, viewed, introspected and interconnected, although this still isn't as easy and performant as a fully relational data model; and (iv) data types can be rapidly expanded as required to meet the demands of new analyses being added to the framework (e.g., adding atom mappings to metabolic models).

The data model in Pathway Tools is completely distinct from the other platforms, maintaining data internally within a relational database, while using primarily custom flat-file-formats that are specific to Pathway Tools for data exchange. This has the advantage of maintaining data in a highly interconnected and queryable format within the Pathway Tools framework. Introspection, search, standardization, and internal consistency are all great strengths of this approach. However, this comes at the cost of granularity in support for versioning, sharing, and provenance in Pathway Tools.

2D. Comparison of Provenance, Data Sharing, and Data Versioning

Our data model comparison reveals how the provenance, data sharing, and data versioning features of our selected frameworks depend significantly on the data model of the framework. Galaxy, CyVerse, BaseSpace, GenePattern and KBase, all of which have file-based or object-based data models, have similar support for provenance and sharing. All of these frameworks maintain information about the input parameters and apps used to produce each object stored in the system (limited in the case of GenePattern). All of these frameworks also support sharing at the level of individual objects. However, this always involves copying objects into “libraries” (Galaxy), “folders” (CyVerse), “projects” (BaseSpace), “notebooks” (GenePattern), or “Narratives” (KBase) and sharing those containers with other users. Galaxy, CyVerse, BaseSpace, GenePattern, and KBase do vary in how they handle versioning. Only KBase maintains an explicit version number of every overwritten object, and only KBase allows an overwritten object to be reverted to a previous version. BaseSpace and GenePattern do not support data versioning, instead relying on users not to overwrite data that they want to keep

Nature Biotechnology: doi:10.1038/nbt.4163

(although this can result in loss of the data needed to repeat a particular downstream result). CyVerse and Galaxy follow a very different approach in that objects are namespaced and timestamped according to the workflow and the previous objects from which they were derived. Thus, it is essentially impossible to overwrite an object, and all versions of all data are preserved unless explicitly deleted by the user.

In this area, Pathway Tools is quite different from the other frameworks, which is not surprising given its very different data model. Versioning in Pathway Tools only occurs at the level of an entire PathwayDB, and sharing is only supported by exporting data into files, which can then be shared offline and imported elsewhere. Pathway Tools only preserves provenance in the annotations and curations made to its underlying relational database, although in this case, provenance can be maintained with a granularity and detail that exceeds the other systems discussed.

2E. Comparison of Built-in Reference Data

Reference data is another area where KBase stands out among the platforms we selected for comparison. Reference data is vital in that it serves to place user data and analysis results into the broader context of all other known data of the same type. It is often more useful to understand how a genome is different from its phylogenetically close neighbors than to understand every single detail of the genome itself.

Among all our platforms selected for comparison, only KBase and Pathway Tools offer their own internally managed, organized, and curated reference data collections. KBase offers a reference database of over 90K microbial and eukaryotic genomes maintained and periodically synchronized with RefSeq and Phytozome. To facilitate comparison, all of the microbial genomes have been annotated using the RAST genome annotation app in KBase. KBase also offers a reference database of biochemistry, including 27K compounds, 34K reactions, and 522 media formulations. The reference genomes in KBase are readily available for copying into any KBase Narrative for analysis and comparison, but they also form the basis for some apps that analyze user data in the context of this reference data (e.g., the *Insert Genomes into Species Tree* app). The reference biochemistry in KBase forms the basis for the metabolic model reconstruction, standardization, and gapfilling tools in KBase.

Pathway Tools offers ready access to a database of 9318 Pathway Genome Databases, each of which represents a single genome, a metabolic reconstruction, and a basic model. Pathway Tools also deeply integrates a reference biochemistry database comprised of 14K reactions and 13K compounds. Even more than KBase, this reference data in Pathway Tools is at the core of every analysis a user does in the platform.

While Galaxy, GenePattern, CyVerse, and BaseSpace do not presently have their own internally managed and organized reference data, it is important to note that: (i) all four of these platforms include apps that facilitate the download of reference data from other existing databases (e.g., NCBI); (ii) all four have public data available for download and access to

varying degree (e.g., CyVerse has a large and diverse *data commons* while BaseSpace has example datasets); and (iii) many apps integrated into these platforms maintain their own sizable reference databases. This last point is critical, and in fact, provides one reason why maintaining an internal reference database is useful. Users will run many different apps on their data, which internally may utilize their own wide range of internal reference data. Problems may arise when mixing and matching apps that rely on different and inconsistent reference data.

Overall, KBase stands out in this category for its breadth of genomic and biochemical reference data.

2F. Comparison of Third-party Development and Custom Code Support

In terms of support for third-party development, KBase, BaseSpace, GenePattern, CyVerse, and Galaxy all offer a similar experience, while Pathway Tools is quite different. In large part, this is due to the fact that KBase, BaseSpace, GenePattern, CyVerse, and Galaxy were all designed in part to serve as platforms for the deployment of third-party apps. All of these platforms have their own equivalent of an app catalog (called the Discovery Environment in CyVerse and the ToolShed in Galaxy). These app catalogs enable users to discover apps, read documentation related to the apps, rate the apps, and view apps created and shared by other users. All of these platforms offer an SDK of some form, and use virtualization technology to simplify deployment (Galaxy, CyVerse, and BaseSpace use Docker just like KBase does). Galaxy, KBase, CyVerse, GenePattern, and BaseSpace enable users to create their own custom UIs for their apps using general spec files encoded in either XML (Galaxy) or JSON (KBase, CyVerse, BaseSpace). The complexity associated with the addition of an app is roughly equivalent in all five platforms. The object-based data model in KBase does create added complexity for developers as they need to convert between objects and files when wrapping a tool, but conversely, these developers also often benefit from the greater standardization of objects fed into their tool on KBase. Galaxy has a lower learning curve and simpler app development process, but app registration system-wide is more difficult. CyVerse, GenePattern, BaseSpace and KBase have steeper learning curves to create a new app, but KBase has the simplest interface for registering, sharing, and maintaining an app in the App Catalog.

One unique aspect of the KBase SDK is the ease with which a developer can programmatically call any KBase app from within another app (e.g., the metabolic model reconstruction app can invoke the species tree building app internally to place a genome in a specific phylogenetic neighborhood). This capability will grow in power and importance as the number of apps available in the KBase app catalog increases.

A second unique aspect of the KBase SDK is the support for binding in a wide range of programming languages, as well as the ability to construct a new module from a standard template in any of the same programming languages. CyVerse, GenePattern, BaseSpace, and Galaxy all offer only REST web services for interacting with the broader platforms (e.g., accessing data).

Pathway Tools also supports third party development, but in a much less formal, flexible, or automated fashion. Pathway Tools offers a LISP command interface, which exposes an API to access Pathway Tools data and run Pathway Tools applications interactively. Users can use this API to create new tools, and they can work with the Pathway Tools developers to get their tool integrated into the platform, but there is no way for a user to do this independently without interacting with core developers. Additionally, the tools must be written, at least in part, in LISP in order to interact with the rest of the Pathway Tools platform. KBase, CyVerse, BaseSpace, and Galaxy all offer language-agnostic REST or JSON RPC interfaces for platform interaction.

2G. Comparison of Custom Code Support within Platform Workflows

One key differentiator of KBase is that it's built on the Jupyter framework, so it enables users to seamlessly integrate IPython code cells into their workflows, either to run KBase apps in bulk, or to implement custom analysis steps that are not yet implemented within an app. Both of these capabilities are used to good effect in the exemplar Narratives described above. We explored in our comparison whether any other selected platforms offer a similar capability.

GenePattern is the most similar platform to KBase in terms of this capability since it is also built on top of the Jupyter framework. GenePattern also allows seamless integration of custom code and Markdown cells into users' workflows, and GenePattern offers a programmatic interface for running apps in the platform.

Neither CyVerse nor BaseSpace exhibit this capacity. The only way to integrate custom code into a workflow in these environments is to create a new app using the SDK. However, CyVerse does allow users to run local scripts in Shell, Perl, Python or R and run basic utilities based on these languages in the Discovery Environment for data/file processing. Users can write custom workflows using the LISP command interface in Pathway Tools, but rapidly sharing these custom workflows with others is difficult, as generally sharing workflows in Pathway Tools is not supported.

Galaxy does not natively support the integration of custom code either. However, there are deploys of Galaxy available within Jupyter, where Python code can be used to run Galaxy apps within Jupyter code cells. This functionality is not nearly as integrated as the code cells in KBase, but it does offer a similar capability.

Overall, a strong argument can be made that this important feature, at least in a fully integrated form, is unique to the KBase and GenePattern platforms. One of the challenges in using any online workflow system for scientific analysis is that virtually every scientific workflow is distinct (given the importance of novel discovery in science). Thus, it is very easy to run into a step in one's workflow for which no app conveniently exists in the platform one is using. When this happens, the user is forced to pull their data out of the platform they are using and load it into another environment where the needed analysis is available. This disrupts the continuity, provenance, and containment of the analysis. The analysis can no longer be shared as a single

self-contained entity for others to run. Thus, support for custom code is a truly important function for this type of platform, and it is distinct to KBase and GenePattern.

2H. Comparison of Available Scientific Functionality

Scientific functionality is one of the areas with the greatest variability among the platforms included in our comparison. Although there are plenty of examples of specific apps that are available in multiple platforms, no two platforms offered the same range of functionality, and all platforms had distinct areas of strength. Table 1 shows approximate support for different types of functionality in each platform. Note that this comparison focuses on functionality relating to the analysis of microbial and plant genomes, as this reflects the mission-space of KBase as a DOE resource.

The app functionality in KBase differs from existing systems in several ways. The seamless integration of code cells that KBase offers is distinctive, but not entirely unique--GenePattern[21] and Synapse offer a similar capability. Galaxy[18], Taverna[24], CyVerse[19], XSEDE[25], myExperiment[26], and GenePattern[21] overlap with many of the bioinformatics workflows in KBase but lack the metabolic modeling capabilities. COBRA Toolbox[30], Pathway Tools[31], and RAVEN Toolbox[32] support metabolic modeling but offer only minimal support for genome sequence analysis. In terms of science functionality, each platform has its own set of strengths and weaknesses. There are many categories where nearly all platforms have at least something to offer (although some have more than others). These include genome assembly, genome annotation, RNA-seq, comparative genomics, expression analysis, and assembly. However, among these categories, some platforms are clear gold standards in certain areas: CyVerse, GenePattern, and Galaxy for RNA-seq, variation, and comparative genomics; and KBase for assembly and annotation.

Finally, there are areas of functionality that really distinguish between platforms because very few platforms offer any functionality at all. In metabolic modeling, only Pathway Tools and KBase offer functionality; in metagenome annotation, only CyVerse and Galaxy offer functionality; and in metabolomics and chemistry, only Galaxy offers functionality. Currently, KBase's capabilities for community model reconstruction, plant model reconstruction, community model gapfilling, and expression data model integration are completely unique to the KBase platform. Unsurprisingly, an examination of the app run counts in KBase reveals that the most runs are applied to the apps where KBase is strongest: annotation, modeling, and assembly

2I. Summary of Platform Comparison

Overall, from this comparison we see that KBase is extremely distinct from other bioinformatics and computational biology platforms that exist today. The data model is a key area of distinction: KBase uses biological types as objects, while most other platforms use files. While this makes file conversion more challenging for developers, it brings the benefit of generally making all tools much more interoperable and integrated, and requiring far fewer apps simply to convert data from one format to another. The KBase data model also permits introspection,

enabling viewers, summary statistics, dropdowns and search utilities that offer views of subobjects within an entity (reactions in a model, genes in a genome).

User experience is another key differentiator in KBase. With its integration on top of the Jupyter framework, KBase offers users the ability to run their analysis workflows within a notebook environment that also supports the seamless integration of custom text, graphics, and even executable code. Users can organize their apps, text, and graphics into rich, reproducible, scientific stories that may be shared and extended by others. Only GenePattern shares this capability with KBase, and GenePattern lacks the tight integration of data into this notebook environment that KBase offers.

Custom code support is another strength for KBase. KBase and GenePattern are the only platforms that offer this capacity natively within their primary interface. This capability is essential, as it enables a user to fill gaps in a workflow by writing custom code directly within the Narrative notebook. This enables a user to maintain the continuity of a Narrative as much as possible by avoiding the need to export data and analyze offline or in another platform in order to complete custom analyses. There is an enormous benefit to reproducibility if all work for a single complex study is performed in a single environment.

Reference data is also a key distinguishing component of the KBase platform. Only Pathway Tools and KBase maintain their own internal curated reference data. This data is crucial for placing user data into context. While other platforms do support reference data used by individual tools, there is great benefit in consolidating reference data for tools at much as possible to ensure that all tools are using a common reference data. The reference data in KBase is also instrumental to planned functionality like the Knowledge Engine.

Finally, scientific functionality is another key area of distinction for KBase. KBase offers diverse functionality that is nearly as broad as Galaxy (in terms of the number of categories of apps available) if not always as deep as Galaxy (in terms of the number of apps in each category). Additionally, there are some areas of functionality where KBase is a gold standard compared with the other platforms in our comparison, including genome assembly, annotation, and metabolic modeling.

This comparison focused on the most distinguishing features of the KBase platform, exploring how these features contrast with other platforms. As a result, this comparison ignores many important features that differentiate these other platforms from KBase. For example, BaseSpace, Galaxy, and CyVerse all excel at the annotation and analysis of human and other eukaryotic genome data. In contrast, the KBase user agreement explicitly prohibits the upload and analysis of human data in KBase. As a further example, Galaxy and Pathway Tools both excel at portability, meaning users can easily install and run their own instance of these systems. In contrast, platforms like KBase, GenePattern, and BaseSpace are fully centralized.

Supplementary Note 3. Code and Data Availability

3A. Code Availability

The KBase code, available at github.com/kbase, is open source and freely distributed under the MIT License. The web-accessible KBase system (narrative.kbase.us) is run on DOE computing infrastructure and is freely available for anyone to use. KBase adheres to the FAIR (Findable, Accessible, Interoperable, Re-usable) data principles endorsed by many funding agencies and scientific organizations[33]

3B. Data Availability

All data generated or analyzed during this study are included in this published article and Supplementary Note 1 as links to the original work, or in the associated KBase Narratives linked here. An earlier version of this paper was published as a preprint[34].

Supplementary Figures

Supplementary Figure 1. Code cell for batch processing. A custom code cell created within the Narrative Interface that constructs CMMs for thousands of genomes.

Construction of Core Metabolic Models in Bulk using a Code Cell # Janaka

```
import time
import pprint
import json
import sys
from biokbase.narrative.jobs.appmanager import AppManager
version = "release"

#Construction of Core Metabolic Models in Bulk using a Code Cell # Janaka Edirisinghe v.o1 03/16/2017

ws = biokbase.narrative.clients.get("workspace")

my_list = ws.list_objects({'ids':['358'],'type':'KBaseGenomes.Genome','minObjectID':0,'maxObjectID':50
count = 0
for genome in my_list:
   #print 'Genome :', genome[1]
   count += 1
   if count < 501:
        #print 'Now processing genome id :', genome[1]

        print 'Starting Core Metabolic construction for the genome ',genome[1]
        genome_ob = ws.copy_object({'from':{'objid':genome[0],'wsid': 358},'to':{'wsid': 20186,'name':
```

Supplementary Figure 2. "Compare Flux with Expression" output. Bar chart produced by the [“Compare Flux with Expression” app] in KBase, which evaluates the reconciliation of metabolic model predictions against expression data. All of the data points are categorized into biochemical pathways.

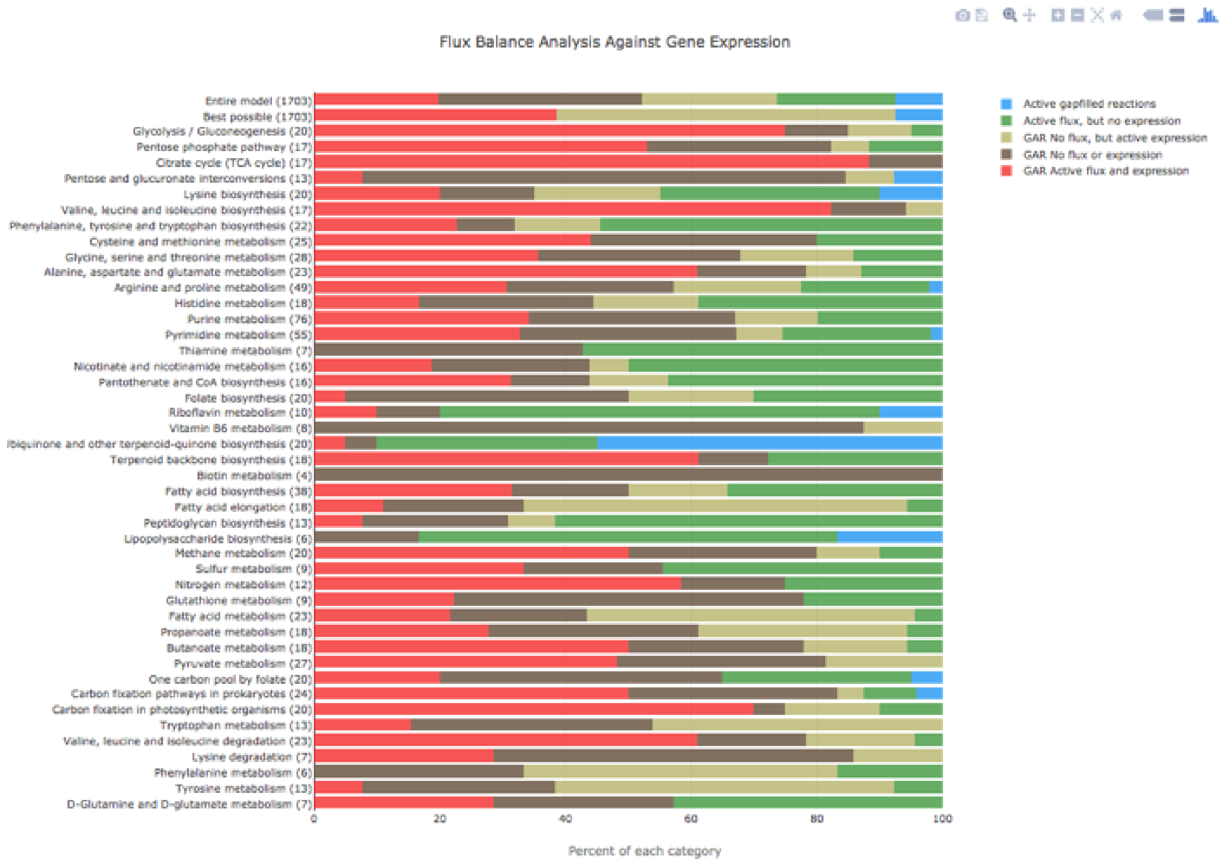

Supplementary Figure 3. Sharing of data, commentary and workflows using KBase Narratives. Two researchers (Alice and Bob) create analysis workflows that complement each other's research resulting in more intuitive and complete scientific conclusions.

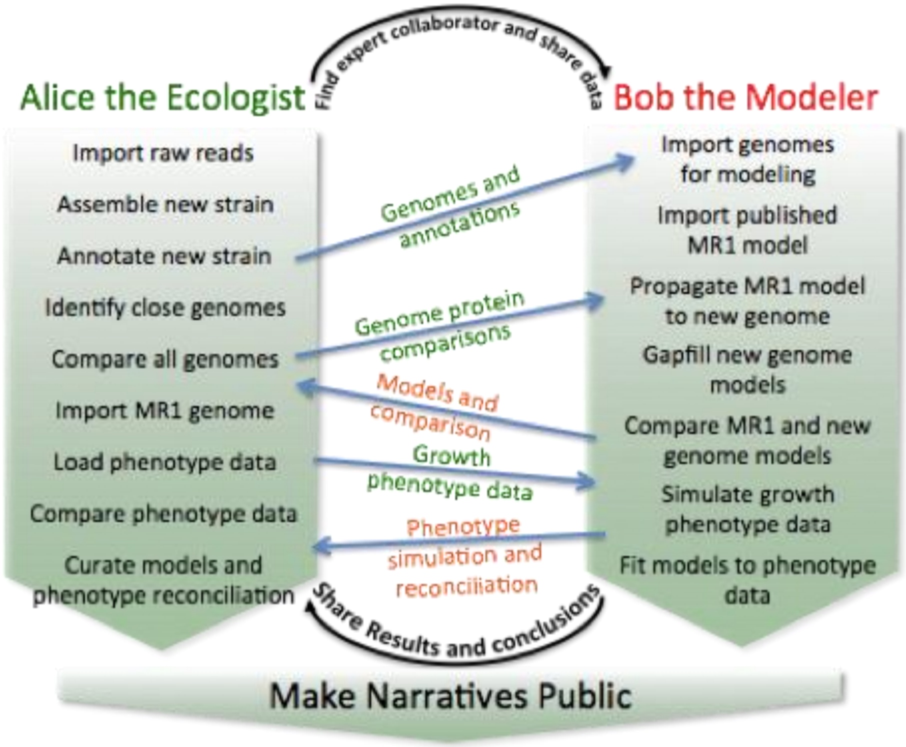

Supplementary Tables

Supplementary Table 1. Comparison of functionality available across evaluated platforms

Category	KBase	CyVerse	BaseSpace	Galaxy	GenePattern	Pathway Tools
**Genome assembly**	High	Medium	Low	Medium	None	None
**Microbial genome annotation**	High	Medium	Low	Low	Low	Low
**Metagenome assembly and contig binning**	Medium	Medium	None	Low	None	None
**Metagenome annotation**	None	Medium	None	Medium	None	None
**Variation and GWAS**	None	High	Medium	High	High	None

**RNA-seq**	Medium	High	Low	High	High	None
**Metabolic modeling and chemistry**	High	None	None	None	Low	High
**Metabolomics and chemistry**	Low	None	None	Medium	None	Low
**Comparative genomics**	Low	High	Low	High	Low	Low
**Expression analysis**	Low	Low	Low	High	High	Low

Supplementary References

1. Edirisinghe, J.N. et al. Modeling central metabolism and energy biosynthesis across microbial life. *BMC Genomics* **17**, 568 (2016).
2. Wu, X. et al. Draft Genome Sequences of Two Janthinobacteriumlividum Strains, Isolated from Pristine Groundwater Collected from the Oak Ridge Field Research Center. *Genome Announc* **5** (2017).
3. Seemann, T. Prokka: rapid prokaryotic genome annotation. *Bioinformatics* **30**, 2068-2069 (2014).
4. Brettin, T. et al. RASTtk: a modular and extensible implementation of the RAST algorithm for building custom annotation pipelines and annotating batches of genomes. *Sci Rep* **5**, 8365 (2015).
5. Li, L., Stoeckert, C.J., Jr. & Roos, D.S. OrthoMCL: identification of ortholog groups for eukaryotic genomes. *Genome Res* **13**, 2178-2189 (2003).
6. Henry, C.S. et al. Microbial community metabolic modeling: A community data-driven network reconstruction. *J Cell Physiol* (2016).
7. Weston, D.J. et al. Sphagnum physiology in the context of changing climate: emergent influences of genomics, modelling and host-microbiome interactions on understanding ecosystem function. *Plant Cell Environ* **38**, 1737-1751 (2015).
8. Wilkinson, M.D. et al. The FAIR Guiding Principles for scientific data management and stewardship. *Sci Data* **3**, 160018 (2016).
9. Wu, Y.W., Tang, Y.H., Tringe, S.G., Simmons, B.A. & Singer, S.W. MaxBin: an automated binning method to recover individual genomes from metagenomes using an expectation-maximization algorithm. *Microbiome* **2**, 26 (2014).
10. Aziz, R.K. et al. The RAST Server: rapid annotations using subsystems technology. *BMC Genomics* **9**, 75 (2008).
11. Price, M.N., Dehal, P.S. & Arkin, A.P. FastTree 2--approximately maximum-likelihood trees for large alignments. *PLoS One* **5**, e9490 (2010).
12. Henry, C.S. et al. High-throughput generation, optimization, and analysis of genome-scale metabolic models. *Nature Biotechnology* **Nbt.1672**, 1-6 (2010).

13. Faria, J.P. et al. Constructing and Analyzing Metabolic Flux Models of Microbial Communities. *Hydrocarbon and Lipid Microbiology Protocols* (2016).
14. Latendresse, M. Efficiently gap-filling reaction networks. *BMC Bioinformatics* **15**, 225 (2014).
15. Dreyfuss, J.M. et al. Reconstruction and validation of a genome-scale metabolic model for the filamentous fungus Neurospora crassa using FARM. *PLoS Comput Biol* **9**, e1003126 (2013).
16. Ghosh, S. & Chan, C.K. Analysis of RNA-Seq Data Using TopHat and Cufflinks. *Methods Mol Biol* **1374**, 339-361 (2016).
17. Faria, J.P. et al. Computing and Applying Atomic Regulons to Understand Gene Expression and Regulation. *Front Microbiol* **7**, 1819 (2016).
18. Goecks, J., Nekrutenko, A., Taylor, J. & Team, G. Galaxy: a comprehensive approach for supporting accessible, reproducible, and transparent computational research in the life sciences. *Genome Biology* **11** (2010).
19. Goff, S.A. et al. The iPlant Collaborative: Cyberinfrastructure for Plant Biology. *Front Plant Sci* **2**, 34 (2011).
20. Karp, P.D. et al. Pathway Tools version 19.0 update: software for pathway/genome informatics and systems biology. *Brief Bioinform* **17**, 877-890 (2016).
21. Reich, M. et al. GenePattern 2.0. *Nat Genet* **38**, 500-501 (2006).
22. Perez, F. & Granger, B.E. IPython: A system for interactive scientific computing. *Computing in Science & Engineering* **9**, 21-29 (2007).
23. Kluyver, T. et al. Jupyter Notebooks—a publishing format for reproducible computational workflows. *Positioning and Power in Academic Publishing: Players, Agents and Agendas*, 87-90 (2016).
24. Oinn, T. et al. Taverna: a tool for the composition and enactment of bioinformatics workflows. *Bioinformatics* **20**, 3045-3054 (2004).
25. Towns, J. et al. XSEDE: Accelerating Scientific Discovery. *Computing in Science & Engineering* **16**, 62-74 (2014).
26. Goble, C.A. et al. myExperiment: a repository and social network for the sharing of bioinformatics workflows. *Nucleic Acids Research* **38**, W677-W682 (2010).
27. Altintas, I. et al. Kepler: An extensible system for design and execution of scientific workflows. *16th International Conference on Scientific and Statistical Database Management, Proceedings*, 423-424 (2004).
28. Deelman, E. et al. Pegasus: A framework for mapping complex scientific workflows onto distributed systems. *Sci Programming-Neth* **13**, 219-237 (2005).
29. Ananthakrishnan, R., Chard, K., Foster, I. & Tuecke, S. Globus platform-as-a-service for collaborative science applications. *Concurrency and Computation-Practice & Experience* **27**, 290-305 (2015).
30. Schellenberger, J. et al. Quantitative prediction of cellular metabolism with constraint-based models: the COBRA Toolbox v2.0. *Nat Protoc* **6**, 1290-1307 (2011).
31. Karp, P.D. et al. The EcoCyc and MetaCyc databases. *Nucleic Acids Research* **28**, 56-59 (2000).
32. Agren, R. et al. The RAVEN toolbox and its use for generating a genome-scale metabolic model for Penicillium chrysogenum. *PLoS Comput Biol* **9**, e1002980 (2013).
33. Wilkinson, M.D. et al. The FAIR Guiding Principles for scientific data management and stewardship. *Sci Data* **3**, 160018 (2016)
34. Arkin, A.P. et al. The DOE Systems Biology Knowledgebase (KBase). *bioRxiv* **preprint first posted online Dec. 22, 2016** (2016).
